# Stereotactic Body Radiotherapy of a Lung Tumor With Large Respiratory Motion Using Real-Time Adaptive Motion Management: A Case Report

**DOI:** 10.7759/cureus.86436

**Published:** 2025-06-20

**Authors:** Xiaoming Chen, Shelly Hayes, Randi Cohen, Charlie Ma

**Affiliations:** 1 Radiation Oncology, Fox Chase Cancer Center, Philadelphia, USA

**Keywords:** adaptive radiation therapy, lung tumor, motion management, robotic stereotactic radiotherapy, stereotactic body radiotherapy (sbrt)

## Abstract

Large respiratory motion presents a challenging issue for stereotactic body radiotherapy (SBRT) treatment of thoracic or abdominal lesions. In clinical practice, it is common to use the internal target volume (ITV) method to account for respiratory motion. This leads to a larger target volume than the real tumor volume. When the tumor motion is large, the ITV-to-tumor volumetric ratio increases significantly, causing a proportionally large amount of normal tissue to receive a high prescription dose. While there exist different approaches to reduce the ITV, adaptive motion management (AMM), e.g., the respiratory synchrony target tracking method used by CyberKnife (CK), presents an interesting one. In this report, we present a lung SBRT case in which the tumor exhibits huge respiratory motion. The elongated shape of the lesion and the motion made the treatment more challenging if a conventional ITV method was used. After being evaluated initially for the conventional ITV method, the patient was selected to be treated under adaptive motion management with CyberKnife. A dosimetric comparison was performed between plans with/without AMM. The results demonstrated the benefits of the application of AMM and may provide useful clinical data for the future practice of treating lesions with large respiratory motion.

## Introduction

Stereotactic body radiotherapy (SBRT) is increasingly used for cancer management as several high-profile randomized SBRT trials have shown encouraging results [1-3]. With high fractional doses, radiation delivery accuracy is crucial. For thoracic or abdominal cancer treatment, respiratory motion presents a challenging issue, and it is a common practice to use the internal target volume (ITV) to account for the motion effect during planning to make sure the lesion receives the prescription dose. This often leads to a larger volume to be irradiated than the real tumor volume. Different motion management techniques have been used to best minimize the ITV, such as abdominal compression (reduces respiration motion) or gated radiation delivery (beam is on only within a designed breathing window but requires good breathing control for patients) [4,5]. These techniques essentially require the target to adapt to the radiation beam. Conversely, if the beam can adapt to the target motion during treatment, we can minimize the motion effect and irradiate less healthy tissue. This could also lead to a better patient experience as less effort is needed for a patient to maintain a specified treatment geometry.

The respiratory synchrony target tracking method used by CyberKnife (CK) is a useful method for real-time adaptive motion management (AMM), in which the radiation beam can adaptively adjust the direction to target the lesion by using a tumor motion model created during a treatment session [6]. In this way, the target will be the gross tumor itself, and no additional motion margin is needed as in the ITV method.

In this report, we describe an interesting lung SBRT case in which the lesion exhibits large breath-induced motion and an unfavorable elongated shape. Using conventional Linac-based SBRT treatment would irradiate a significant portion of the healthy lung volume and chest wall in order to guarantee sufficient dose coverage for the target, which includes the motion margin. Using the AMM (i.e., respiratory synchrony tumor tracking method) allows us to reduce the volume of the target to be irradiated significantly, as well as to the nearby critical organs. The dosimetric comparison between these two methods will be presented.

## Case presentation

**Figure 1:**
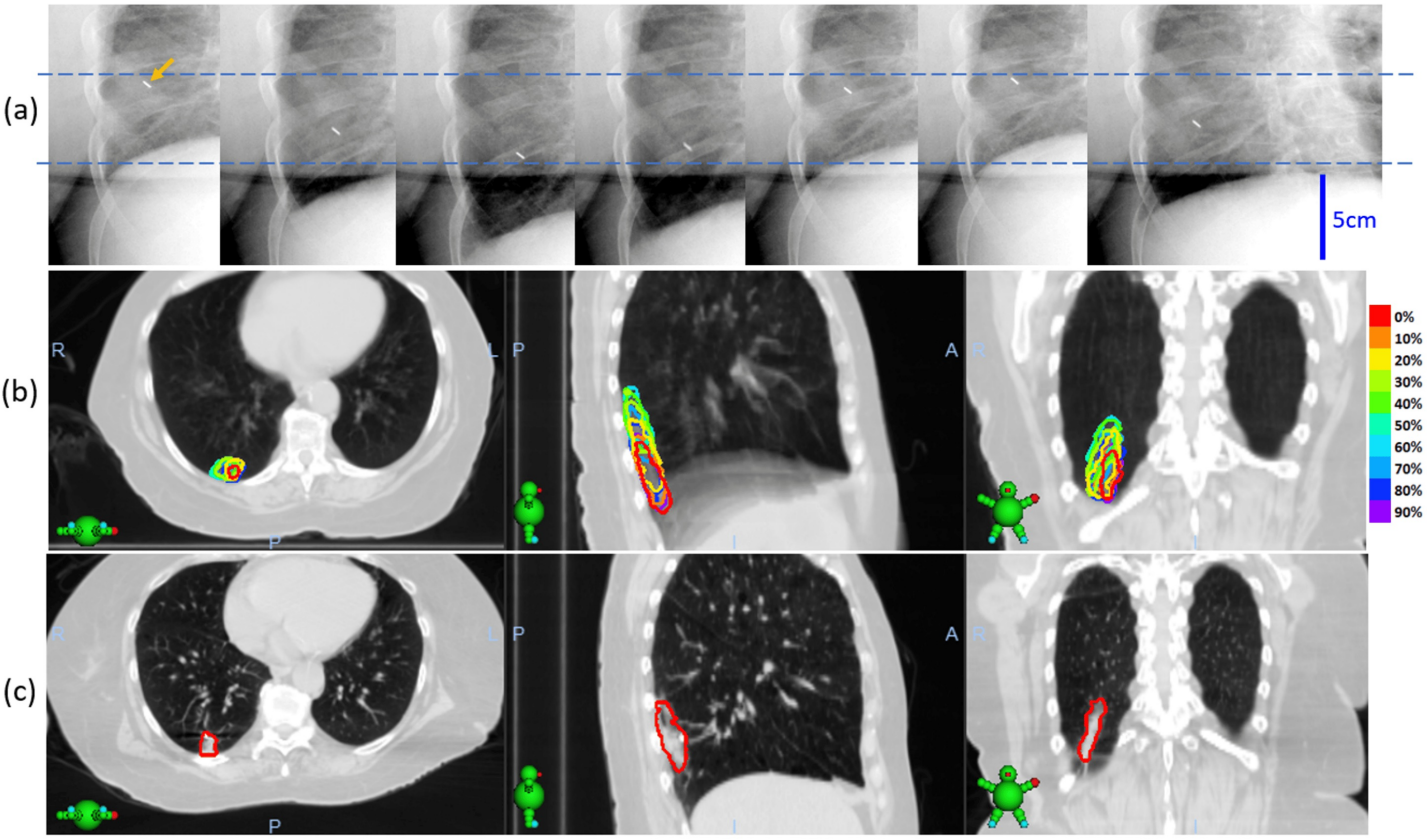
(a) Large breath-induced motion (~5 cm) of lung tumor observed on a series of kV images. The tumor has an implanted gold fiducial marker. (b) Tumor motion trajectory acquired using 4D-CT. The tumor was delineated separately for each respiratory phase, with each phase represented by a different color, as indicated in the color bar. (c) Real tumor shape and size delineated on breath-hold CT scans. 4D-CT: four-dimensional computed tomography, CT: computed tomography

A 72-year-old female patient presented for consideration of radiotherapy to treat a primary cancer in the right lower lobe of the lung with a diagnosis of non-small cell lung cancer (NSCLC) adenocarcinoma (cT1b (2.0 cm) cN0 M0, stage IA2) in 2022. She was initially planned for Linac-based SBRT, and a CT simulation with 4D-CT scan was performed for respiration motion analysis and lesion's ITV creation. However, the 4D-CT showed large breath-induced tumor motion, which had a range of approximately 5 cm in the superior-inferior (S-I) direction (Figure 1). The lesion had an elongated shape measuring approximately 6.2 × 2.0 × 1.2 cm and was located posteriorly abutting the chest wall. By including the motion effect, the resulting ITV had a length of approximately 13.0 cm, and the planning target volume (PTV) had a significant overlap with the chest wall. The initial Linac-based plan prescribed at 50 Gy in five fractions showed that the chest wall dose far exceeded the mandatory criteria limit (outlined in the next section). A decision was made to switch the treatment to CyberKnife with real-time motion management to minimize the PTV.

Treatment planning and delivery

The patient had one gold fiducial marker placed in the lesion about one week before her scheduled simulation for CyberKnife SBRT planning. For simulation, a custom immobilization device was created for the patient while lying supine with arms down. End-expiration breath-hold CT scans were obtained. The gross tumor volume (GTV) was delineated, and a 3 mm margin expansion was used to create the PTV. Nearby organs-at-risk (OARs), such as the lungs, ribs, chest wall, and spinal cord, were also contoured.

A dose of 50 Gy in five fractions was prescribed to cover at least 95% of the PTV. The following dose constraints were required for the OARs: chest wall, V37.5Gy < 30 cc; ribs, Dmax < 57 Gy and V45Gy < 5cc; total lungs (excluding PTV), V13.5Gy < 37% and V (<12.5 Gy) > 1,500 cc; and spinal cord, Dmax < 28 Gy and V22Gy < 0.35 cc [7].

The CyberKnife treatment plan was optimized and finalized in the MultiPlan system (Accuray Inc., Sunnyvale, CA). The plan included a total of 152 beams on 40 nodes and an estimated delivery time of 46 minutes. The final dose was calculated using the Monte Carlo algorithm with a 0.8% uncertainty. For comparison, a separate Linac-based SBRT plan was also generated using the volumetric modulated arc therapy (VMAT) technique for a PTV expanded 5 mm from the ITV, which was created from the 4D-CT scan. The same clinical goals and OAR dose constraints were applied.

Tumor motion was managed by using the real-time respiratory tracking and compensation system, i.e., CyberKnife's synchrony method. The synchrony method uses external markers coupled with an in-room kV imaging system to create a target respiratory motion model. The beam delivery system uses the motion model to guide the robotic arm to adaptively adjust the radiation beam direction in real time so that the beam always aligns with the target. With this synchrony method, the tumor motion can be tracked and radiation dose can be efficiently delivered to the tumor while avoiding unnecessary dose to the nearby healthy tissue.

Results

**Figure 2:**
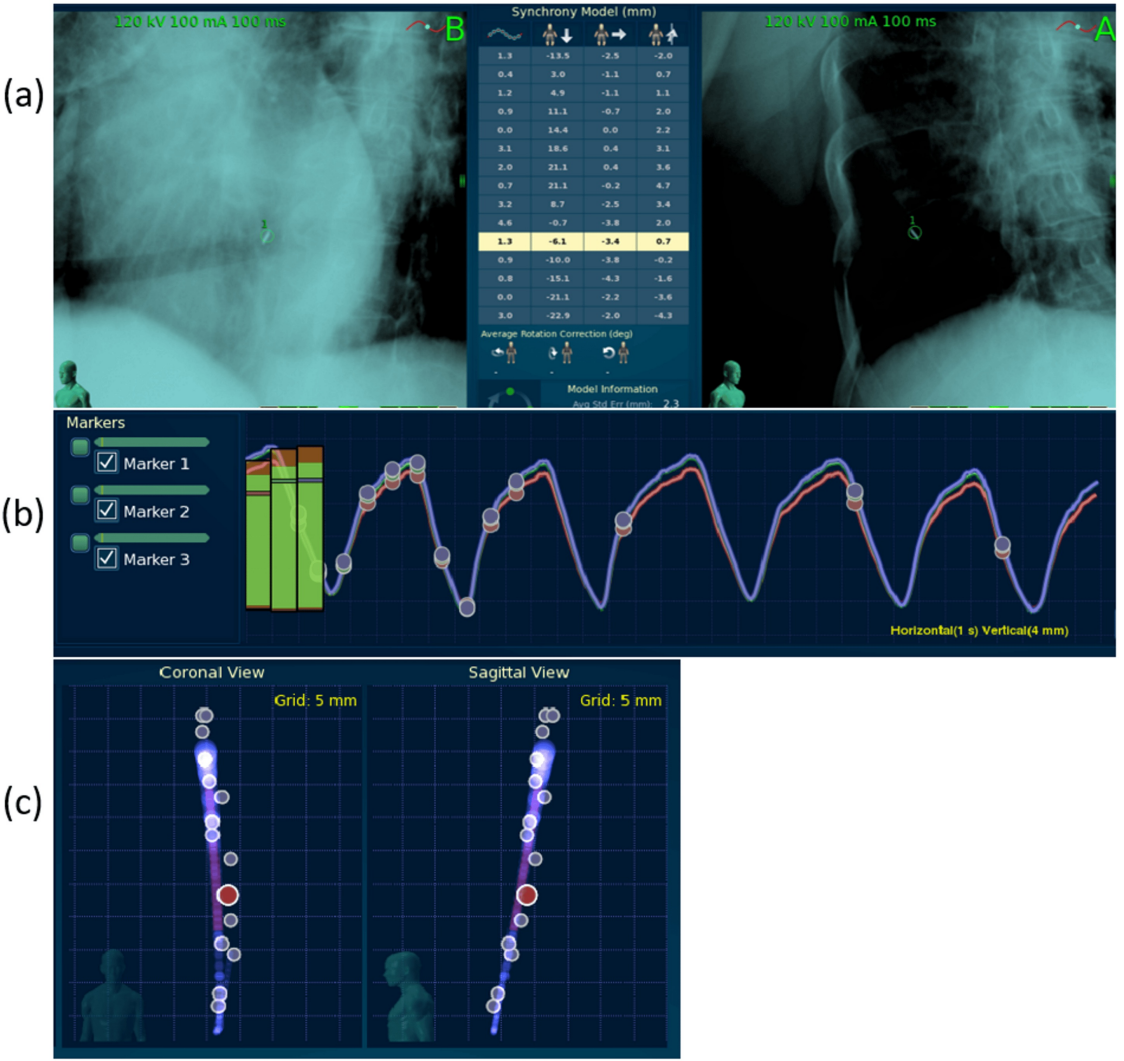
(a) Paired kV images acquired to identify the tumor (fiducial) position. (b) Breathing curve monitored using external markers. The dots on the curve indicate the time and respiratory phase at which the internal tumor (fiducial) position was captured by using the paired kV images as shown in (a). (c) Respiratory synchrony tumor motion model that correlated the external markers' signal to the internal tumor position. The dots represent the tumor positions captured at time points and respiratory phases as shown in (b). The red dot denotes the most recent position.

The patient received the five-fraction treatment as scheduled with no acute treatment toxicity. Figure 2 shows a synchrony tumor motion model created daily during treatment. A series of paired kV images was acquired before radiation delivery to capture the internal tumor/fiducial positions. These kV images were acquired at the peak and valley of the breathing curve, plus several middle points, so that the entire breathing cycle could be modeled. This model was updated continuously during the treatment.

**Figure 3:**
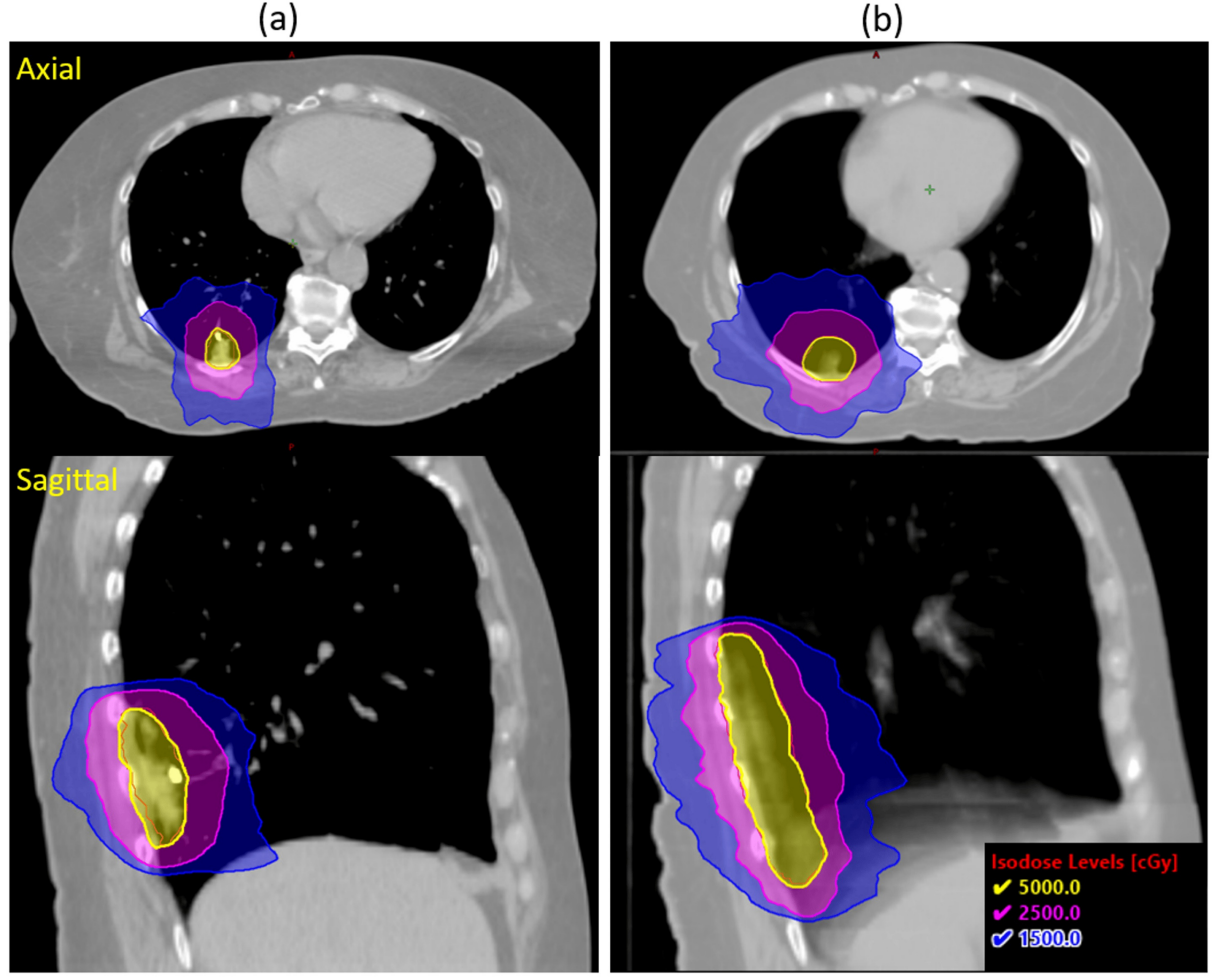
Dose distribution of treatment plan (a) with adaptive motion management, i.e., CyberKnife synchrony method, and (b) without adaptive motion management, i.e., ITV method with full breathing phases. ITV: internal target volume

Figure 3 shows the dose distribution at different isodose lines (IDLs) for the treatment plan with or without AMM. It clearly showed that the Linac-based treatment without AMM will treat a much greater target volume and include more chest wall and normal lung volume to be irradiated.

**Figure 4:**
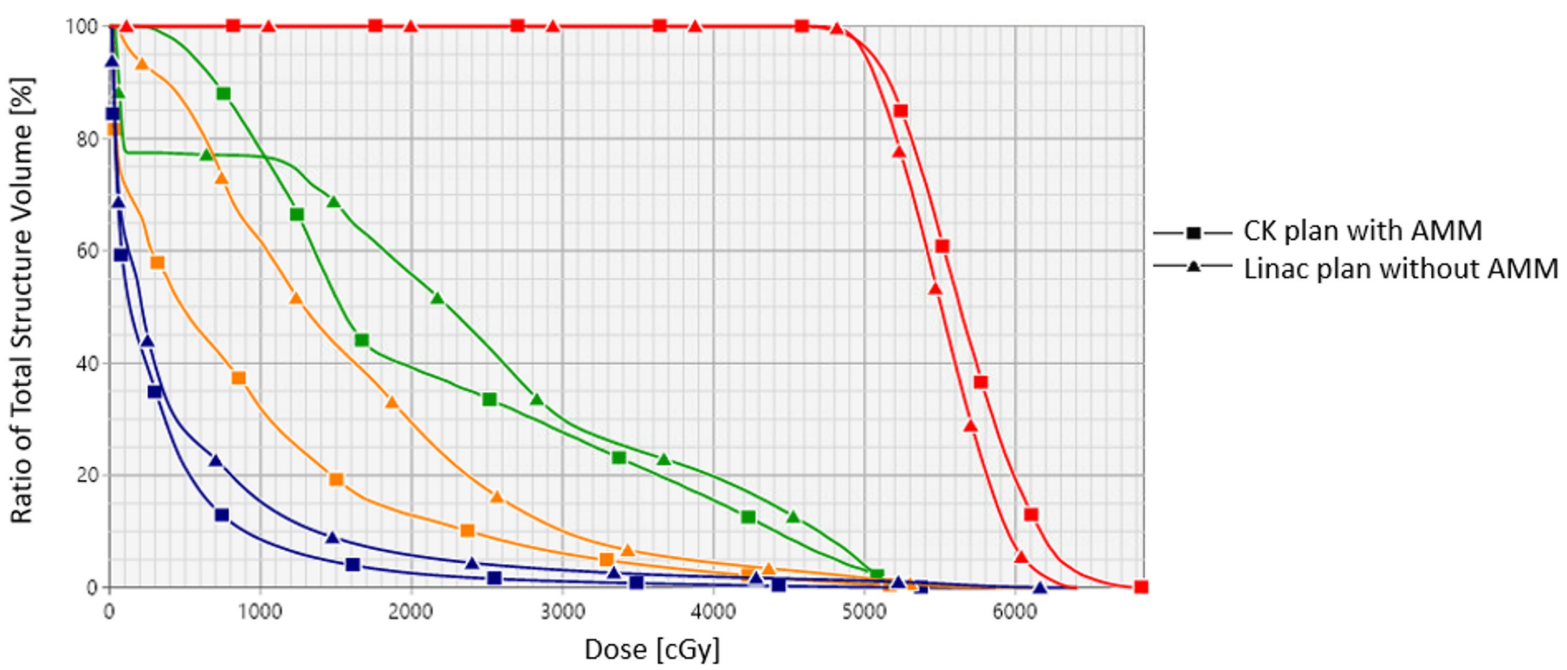
DVH comparison of CK plan with AMM and Linac-based plan without AMM. Red: PTV, green: ribs, orange: chest wall, blue: total lungs DVH: dose-volume histogram, CK: CyberKnife, AMM: adaptive motion management, PTV: planning target volume

The dose-volume histogram (DVH) comparison between the CK plan and Linac-based VMAT plan is shown in Figure 4. The CK plan with AMM had much better normal tissue protection as OARs such as the total lungs, chest wall, and ribs all received much less dose than the VMAT plan without AMM.

**Figure 5:**
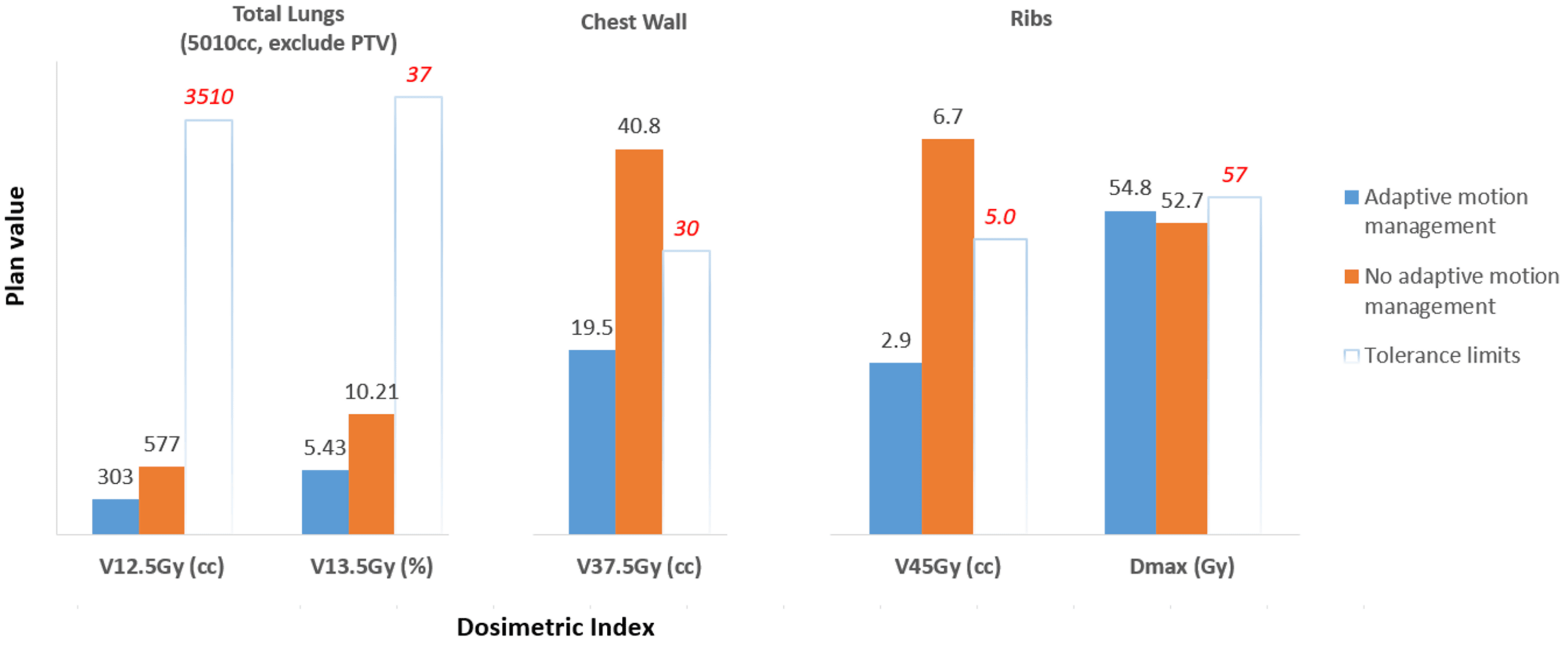
Results of dosimetric index for OARs for AMM (CK plan) and non-AMM (Linac-based plan using ITV method). OARs: organs at risk, AMM: adaptive motion management, CK: CyberKnife, ITV: internal target volume

The results of the dosimetric index for clinical evaluation are shown in Figure 5. For lungs, although both plans met the criteria, the CK plan with AMM had much less volume (approximately 50%) receiving critical doses of 12.5 Gy and 13.5 Gy than the VMAT plan, meaning more normal lung volumes were protected. For the chest wall, the VMAT plan could not meet the criteria for the volume receiving a critical dose of 37.5 Gy or higher, and it was almost two times higher than the CK plan. For the ribs, both plans met the maximal dose limit, but the VMAT plan could not meet the V45Gy criteria, and again, it had approximately two times the volume receiving 45 Gy or higher than the CK plan.

## Discussion

We describe a lung SBRT case treated with adaptive motion management using the CyberKnife. Typically, a lesion in the lower lung has an S-I motion amplitude of 1-2 cm with normal breathing [8,9]. This case shows approximately a 5 cm S-I motion. If treated using the conventional ITV method, a significantly higher volume of OARs would be irradiated. The large breath-induced target motion, plus the elongated shape of the tumor, makes the ITV method unfavorable as significant amounts of lungs and chest wall would receive a high dose in order to cover the moving target. The AMM allows the removal of the motion margin from the planning target and allows beams to only target the tumor itself, thus significantly avoiding unnecessary dose to healthy tissues.

In clinical practice, it is common to treat patients using the conventional ITV method without AMM, and treatment plans can still meet the dosimetric criteria for OARs. However, incorporating AMM allows us to adhere more closely to the ALARA principle (As Low As Reasonably Achievable) and may provide additional benefits to patients. Cases like this may benefit most from AMM, as otherwise, a substantial portion of the chest wall would be irradiated, thereby increasing the risk of radiation-induced chest wall pain [10-12].

It should be noted that the CK treatment took a longer radiation delivery time (~46 minutes in this case) than the Linac-based VMAT plan. However, with AMM, the radiation beam was adaptively adjusted to the target position and requires less patient effort for alignment, unlike the Linac-based treatment, where the patient must make an effort to align to the beam. This may lead to a better patient experience.

## Conclusions

We report an application of AMM for treating a lung tumor with large breath-induced motion, which otherwise will result in a significant unnecessary dose to the normal tissue under conventional Linac-based SBRT. The case highlights the benefits of using AMM for treating a tumor with unfavorable respiratory motion. Similar applications would also be beneficial to liver or abdominal cases where lesions are close to the diaphragm and have significant motion or are surrounded by low-dose-tolerance OARs such as the duodenum and small bowel. With AMM, the removal of the motion margin in the planning target may significantly improve the protection of these OARs.

## References

[1] Katoh N, Soda I, Tamamura H (2017). Clinical outcomes of stage I and IIA non-small cell lung cancer patients treated with stereotactic body radiotherapy using a real-time tumor-tracking radiotherapy system. Radiat Oncol.

[2] Lee P, Loo BW Jr, Biswas T (2021). Local control after stereotactic body radiation therapy for stage I non-small cell lung cancer. Int J Radiat Oncol Biol Phys.

[3] von Reibnitz D, Shaikh F, Wu AJ (2018). Stereotactic body radiation therapy (SBRT) improves local control and overall survival compared to conventionally fractionated radiation for stage I non-small cell lung cancer (NSCLC). Acta Oncol.

[4] Brandner ED, Chetty IJ, Giaddui TG, Xiao Y, Huq MS (2017). Motion management strategies and technical issues associated with stereotactic body radiotherapy of thoracic and upper abdominal tumors: a review from NRG Oncology. Med Phys.

[5] Keall PJ, Mageras GS, Balter JM (2006). The management of respiratory motion in radiation oncology report of AAPM Task Group 76. Med Phys.

[6] Ozhasoglu C, Saw CB, Chen H (2008). Synchrony--cyberknife respiratory compensation technology. Med Dosim.

[7] Timmerman R (2022). A story of hypofractionation and the table on the wall. Int J Radiat Oncol Biol Phys.

[8] Langen KM, Jones DT (2001). Organ motion and its management. Int J Radiat Oncol Biol Phys.

[9] Seppenwoolde Y, Shirato H, Kitamura K (2002). Precise and real-time measurement of 3D tumor motion in lung due to breathing and heartbeat, measured during radiotherapy. Int J Radiat Oncol Biol Phys.

[10] Mutter RW, Liu F, Abreu A, Yorke E, Jackson A, Rosenzweig KE (2012). Dose-volume parameters predict for the development of chest wall pain after stereotactic body radiation for lung cancer. Int J Radiat Oncol Biol Phys.

[11] Welsh J, Thomas J, Shah D (2011). Obesity increases the risk of chest wall pain from thoracic stereotactic body radiation therapy. Int J Radiat Oncol Biol Phys.

[12] Din SU, Williams EL, Jackson A (2015). Impact of fractionation and dose in a multivariate model for radiation-induced chest wall pain. Int J Radiat Oncol Biol Phys.

